# Detection of early relapse in multiple myeloma patients

**DOI:** 10.1186/s13008-025-00143-3

**Published:** 2025-01-29

**Authors:** Tereza Růžičková, Monika Vlachová, Lukáš Pečinka, Monika Brychtová, Marek Večeřa, Lenka Radová, Simona Ševčíková, Marie Jarošová, Josef Havel, Luděk Pour, Sabina Ševčíková

**Affiliations:** 1 Babak Myeloma Group, Department of Pathophysiology, Faculty of Medicine, Masaryk University, Brno, Czech Republic; 2 Department of Internal Medicine, Hematology and Oncology, University Hospital Brno, Brno, Czech Republic; 3 Research Centre for Applied Molecular Oncology (RECAMO), Masaryk Memorial Cancer Institute, Brno, Czech Republic; 4 International Clinical Research Center, St. Anne’s University Hospital Brno, Brno, Czech Republic; 5 Centre for Molecular Medicine, Central European Institute of Technology, Masaryk University, Brno, Czech Republic; 6 Department of Chemistry, Faculty of Science, Masaryk University, Brno, Czech Republic; 7 Department of Clinical Hematology, University Hospital Brno, Brno, Czech Republic

**Keywords:** Multiple myeloma, Liquid biopsy, Relapse, microRNA, MALDI-TOF MS, Small RNA seq, Machine learning

## Abstract

**Background:**

Multiple myeloma (MM) represents the second most common hematological malignancy characterized by the infiltration of the bone marrow by plasma cells that produce monoclonal immunoglobulin. While the quality and length of life of MM patients have significantly increased, MM remains a hard-to-treat disease; almost all patients relapse. As MM is highly heterogenous, patients relapse at different times. It is currently not possible to predict when relapse will occur; numerous studies investigating the dysregulation of non-coding RNA molecules in cancer suggest that microRNAs could be good markers of relapse.

**Results:**

Using small RNA sequencing, we profiled microRNA expression in peripheral blood in three groups of MM patients who relapsed at different intervals. In total, 24 microRNAs were significantly dysregulated among analyzed subgroups. Independent validation by RT-qPCR confirmed changed levels of miR-598-3p in MM patients with different times to relapse. At the same time, differences in the mass spectra between groups were identified using matrix-assisted laser desorption/ionization time of flight mass spectrometry. All results were analyzed by machine learning.

**Conclusion:**

Mass spectrometry coupled with machine learning shows potential as a reliable, rapid, and cost-effective preliminary screening technique to supplement current diagnostics.

**Supplementary Information:**

The online version contains supplementary material available at 10.1186/s13008-025-00143-3.

## Background

Multiple myeloma (MM) is a complex hematological malignancy classified as a monoclonal gammopathy, with an estimated global incidence of 160,000 new cases per year [1, 2]. It is characterized by the uncontrolled proliferation of malignant plasma cells (PCs) and excessive secretion of monoclonal immunoglobulin (Ig) [2]. For MM diagnosis, two criteria are required: the presence of 10% or more clonal PCs in the bone marrow (BM) or a biopsy-proven plasmacytoma. The second criterion is the presence of at least one myeloma-defining events, which include so-called CRAB symptoms (hyper**c**alcemia, **r**enal insufficiency, **a**nemia, and **b**one lesions) and three specific biomarkers: the presence of > 60% clonal PCs in BM, serum free light chain (FLC) ratio ≥ 100 and the detection of more than one focal lesion on MRI [3, 4]. MM treatment options have improved noticeably in the past few decades. New therapies, including monoclonal and bispecific antibodies and CAR-T cell therapy, have extended the overall survival of patients and improved their quality of life [5–7].

Nonetheless, most MM patients relapse [8]. In MM, relapse is defined as ≥ 25% increase from the lowest confirmed serum and/or urine monoclonal Ig value. In unmeasurable cases, it is defined by an increase in BM PC mass of ≥ 10% regardless of baseline. Additional criteria may include a new lesion, an increase in the number of bone lesions, etc [9–11]. Relapse poses a clinical challenge due to MM heterogeneity, new mutations, and drug resistance, making it difficult to determine its timing and severity [12, 13]. Accurate and minimally invasive biomarkers that enable more frequent monitoring of disease become increasingly important.

MicroRNAs (miRNAs), short non-coding RNAs involved in post-transcriptional gene regulation, play essential roles in various physiological processes, including proliferation, apoptosis, and differentiation, all of which are dysregulated in MM [13, 14]. MiRNA dysregulation was implicated in MM pathogenesis, disease progression, and drug resistance [15–17]. MiRNAs are also actively released from cells into body fluids as so-called circulating miRNAs [18, 19]. Unlike other RNA species, circulating miRNAs are stable and resistant to enzymatic degradation; thus, they can be used as biomarkers [20]. Circulating miRNAs from PB could represent a less invasive approach than BM biopsy.

Matrix-assisted laser desorption ionization time-of-flight mass spectrometry (MALDI-TOF MS) allows high-throughput, sensitive detection of molecular patterns [21–23]. This method has been utilized in a combined approach with machine learning (ML) algorithms to study monoclonal gammopathies, revealing molecular signatures relevant to diagnosis, prognosis, and treatment response [24–29].

In this study, we investigated biomarkers for early relapse in MM. While small RNA seq showed the best results, using MALDI-TOF MS and clinical data analyzed by predictive ML models provides economically sound and accurate alternatives for early relapse detection.

## Results

### Small RNA sequencing of microRNAs

Small RNA seq was performed using PB serum samples of 8 MM patients from group A, 8 MM patients from group B, and 8 MM patients from group C. Two samples (one from group A and one from group B) were excluded due to a small number of reads. Sequencing data analysis identified 748 miRNAs. Further analysis showed 360 miRNAs with more than 1 read per million in at least 7 samples.

In total, 8 different miRNAs (miR-16-2-3p, miR-148a-3p, miR-185-5p, miR-335-3p, miR-485-3p, miR-598-3p, miR-4433b-5p, miR-5010-5p) were dysregulated among all three analyzed subgroups (*P* < 0.1). However, the groups did not cluster properly (data not shown). When comparing groups A vs. B, and groups B vs. C, no statistically significant miRNAs were found (data not shown).

**Fig. 1:**
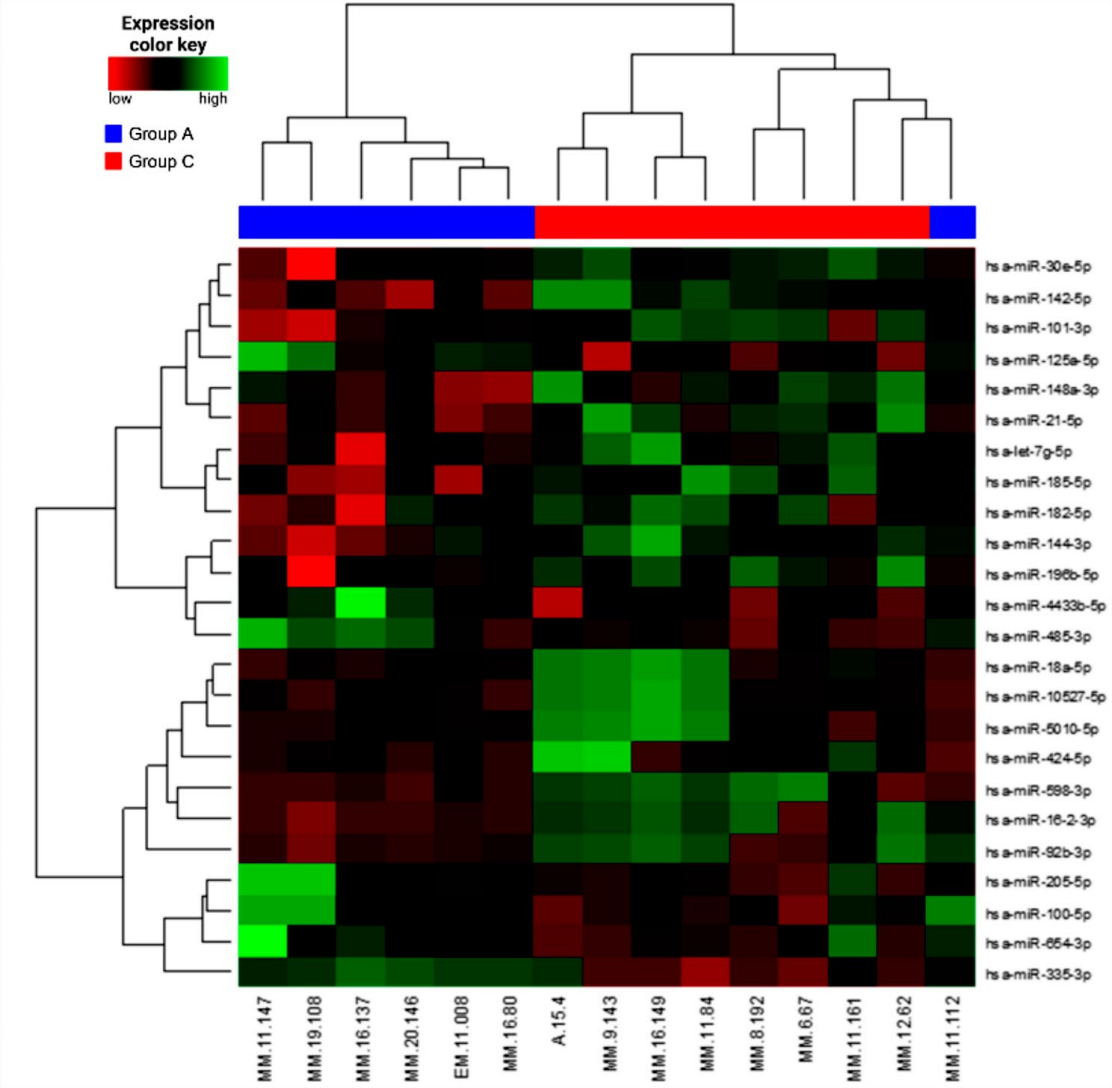
Clustergram and heatmap visualizing 24 miRNAs differentially expressed among 16 multiple myeloma patients, including 7 from group A (relapse within 6 months) (blue) and 8 from group C (relapse after more than 5 years) (red) (adjusted *P* < 0.1) in the exploration phase of the study

The largest differences were found between group A and group C: 24 miRNAs were identified as differentially expressed with *P* < 0.1 (Fig. 1); out of these, miR-16-2-3p and miR-598-3p were the most significantly differentially expressed (*P* < 0.05). Three miRNAs (miR-16-2-3p, miR-92b-3p, miR-598-3p) were chosen for validation in the second step of the study based on the corresponding log-fold change of expression (log-FC) and P-value (Table 1).

**Table 1:** Three most significantly dysregulated miRNAs identified in the exploration phase of the study

miRNA	logFC A vs. C	Adjusted *P*-value
miR-16-2-3p	4.429	0.045
miR-92b-3p	5.231	0.067
miR-598-3p	4.723	0.003

**Fig. 2:**
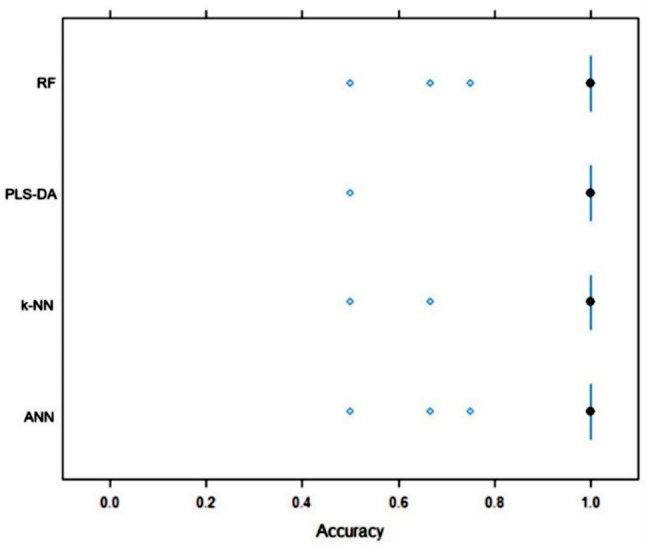
Comparison of 24 differentially expressed miRNAs based on accuracy. Comparing group A (relapse within 6 months) vs. group C (relapse after more than 5 years). The boxes represent the interquartile accuracy range, with black dots indicating the mean value. Whiskers show the accuracy range excluding outliers, which are plotted as blue dots

**Fig. 3:**
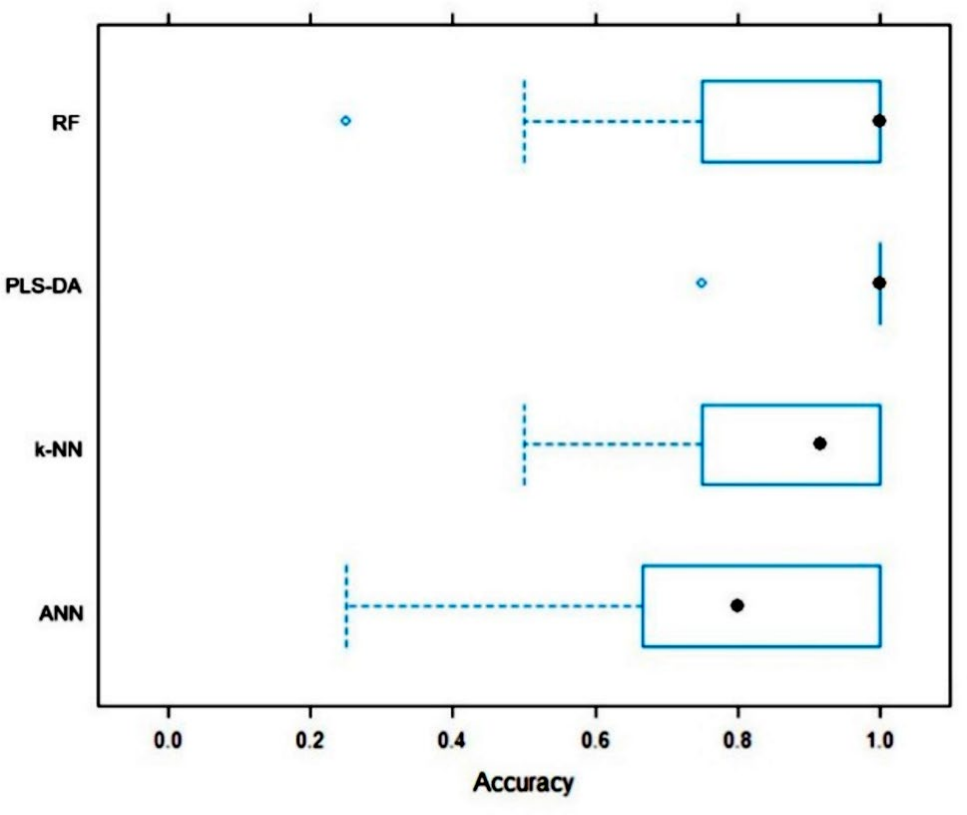
Comparison of 24 differentially expressed miRNAs based on accuracy. Comparing group A (relapse within 6 months) vs. group B (relapse between 12 to 30 months) vs. group C (relapse after more than 5 years). The boxes represent the interquartile accuracy range, with black dots indicating the mean value. Whiskers show the accuracy range excluding outliers, which are plotted as blue dots

Consecutively, the study employed four ML algorithms to predict early relapse in MM patients, namely: PLS-DA (partial least squares discriminant analysis), k-NN (k-nearest neighbors), RF (random forest), and ANN (artificial neural network). These analyses were performed using 8 most dysregulated miRNAs between all 3 groups, 24 mostly dysregulated miRNAs between groups A and C, and 360 miRNAs with more than 1 read per million in at least 7 samples. Evaluation of the models was based on their overall accuracy in predicting the outcome. The best performance was observed when using 24 miRNAs dysregulated between groups A and C. The PLS-DA and RF algorithms exhibited the best performance when comparing groups A and C: PLS-DA: 97.7% (93.3–100%), RF: 92.8% (88.4–97.3%) (Fig. 2) as well as all 3 groups: PLS-DA: 96.8% (94.1–99.5%), RF: 84.8% (79.7–89.9%) (Fig. 3).

### Validation of microRNAs dysregulated among the diagnostic subgroups

**Fig. 4:**
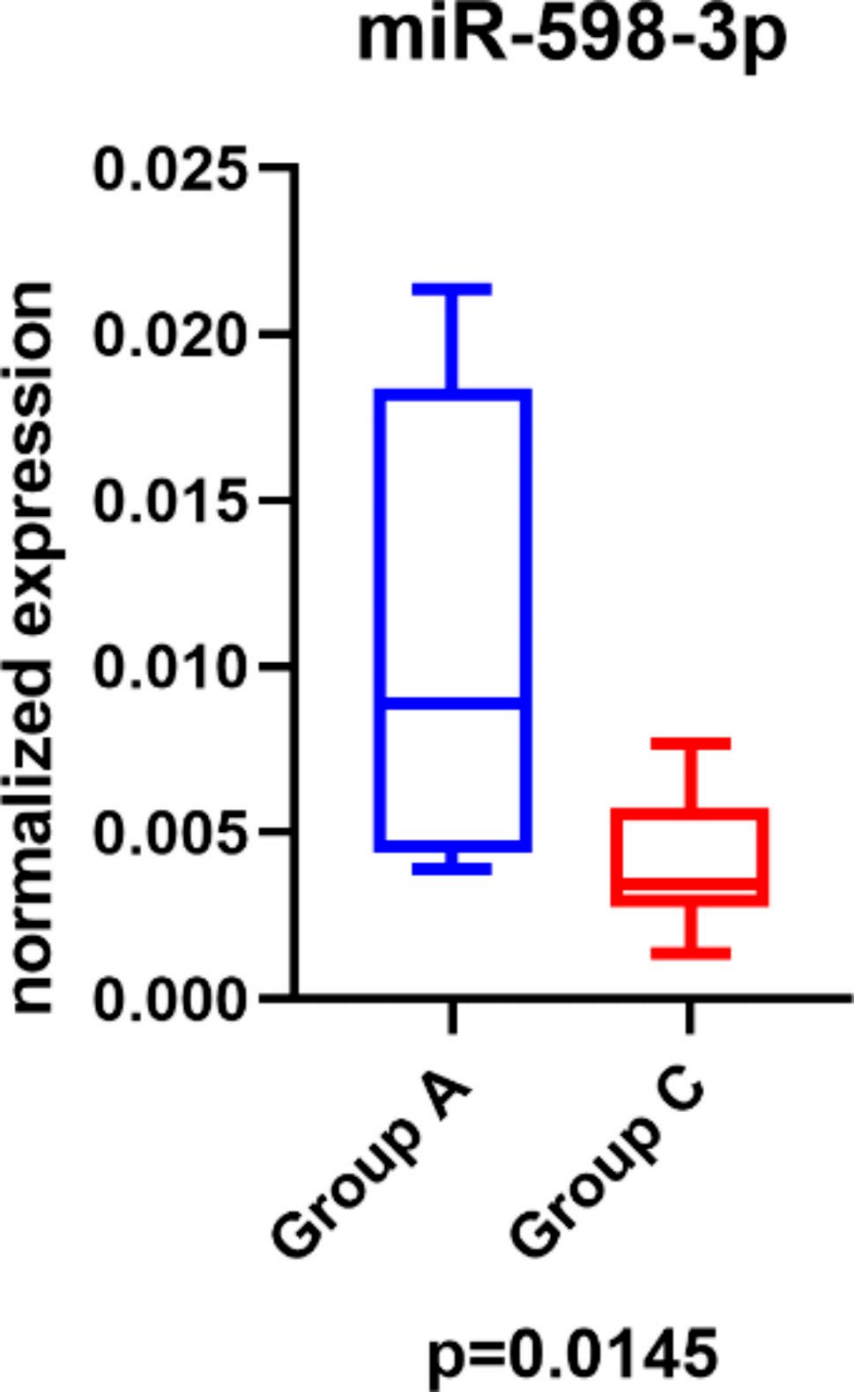
Significantly dysregulated miR-598-3p between group A (relapse within 6 months) (blue) and C (relapse after more than 5 years) (red) (*P* = 0.0145)

Three miRNAs, which were the most dysregulated between group A and group C, were selected for the validation phase of the study. Based on the results of the Mann-Whitney test, significant differences in expression were observed in the case of miR-598-3p (*P* = 0.0145, Fig. 4). Table 2 summarizes the statistical results of the normalized expression values. The expression levels of miR-16-2-3p and miR-92b-3p did not differ between the analyzed subgroups (*P* = 0.2268 and *P* = 0.7108, respectively).

**Table 2:** RT-qPCR validation of miRNAs dysregulated between groups a and C

miRNA	A Median (min-max)	C Median (min-max)	*P*-value
miR-16-2-3p	0.119 (0.020–0.408)	0.055 (0.022–0.25)	0.2268
miR-92b-3p	17.269 (5.161–95.547)	19.450 (6.284–73.665)	0.7108
miR-598-3p	0.005 (0.001–0.021)	0.004 (0.000–0.008)	**0.0145**

Group A, relapse within 6 months; group C, relapse after more than 5 years

**Fig. 5:**
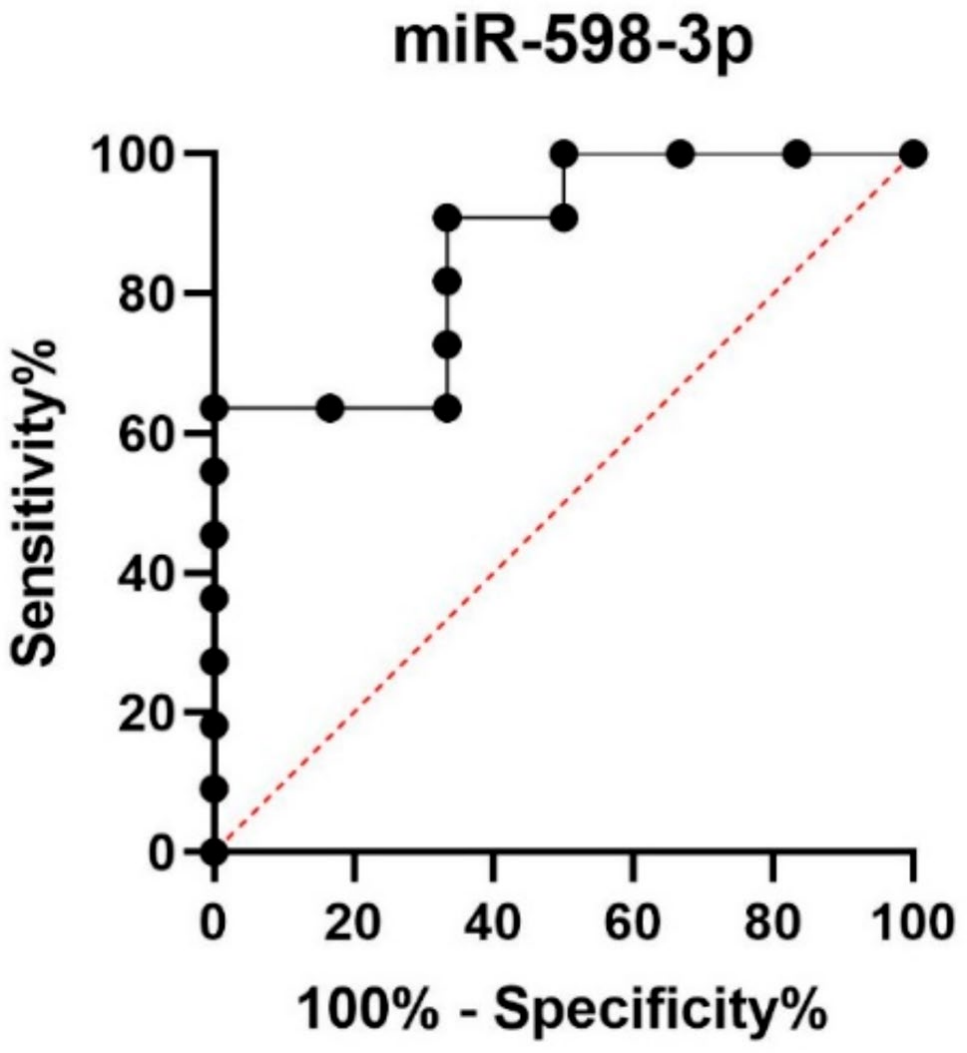
ROC analysis of miR-598-3p (AUC = 0.8636, sensitivity 64%, specificity 100%, cut-off: 0.03724)

Based on the results of ROC analysis, miR-598-3p (AUC = 0.8636, sensitivity 64%, specificity 100%, cut off: 0.03724; Fig. 5) distinguished groups A and C with high specificity.

Correlation analysis was performed between miRNA levels and quantitative clinical characteristics of MM patients. Elevated levels of β2-microglobulin were observed in patients with upregulation of miR-16-2-3p and miR-92b-3p. Other correlation analyses did not show any significant results (Supplementary Table 4, Additional file 1).

### MALDI-TOF MS

**Fig. 6:**
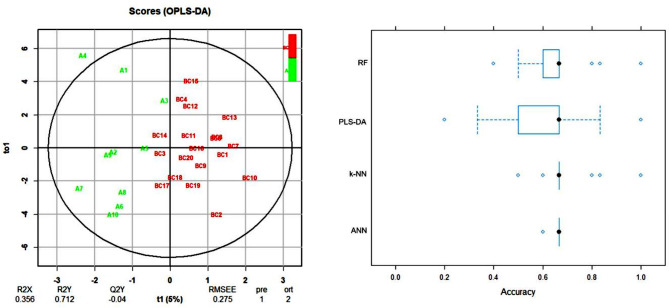
OPLS-DA score plot (left) of analysis of mass spectral data from serum samples. Comparison of established classifiers based on accuracy (right). The boxes represent the interquartile accuracy range, with black dots indicating the mean value. Whiskers show the accuracy range excluding outliers, which are plotted as blue dots

MALDI-TOF MS analysis was performed on a total of 29 PB serum samples, including 10 samples from group A and 19 samples from group BC (combined groups B and C). The mass spectral data were processed using the OPLS-DA method to evaluate whether molecular profiles could distinguish between patient groups based on relapse timing. The optimal number of components to be input to OPLS-DA was determined based on the R2Y and Q2Y values to prevent the overfitting of the model. Consecutively, four ML algorithms (PLS-DA, k-NN, RF, and ANN) were applied to the mass spectral data to verify whether the classification of the selected sample groups can be predicted solely based on computationally processed data (Fig. 6).

**Fig. 7:**
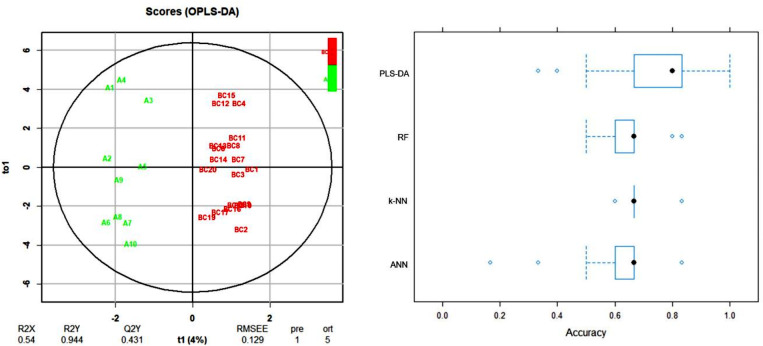
OPLS-DA score plot (left) of analysis of mass spectral data combined with clinical parameters from serum samples. Comparison of established classifiers based on accuracy (right). The boxes represent the interquartile accuracy range, with black dots indicating the mean value. Whiskers show the accuracy range excluding outliers, which are plotted as blue dots

To improve the model predictive value, clinical parameters were added to the dataset; the analyses were performed again, resulting in better-separated clusters and higher accuracy with which the two groups can be distinguished (Fig. 7). The PLS-DA and RF algorithms exhibited the best performance when comparing MALDI data alone: PLS-DA: 62.5% (57.7–67.4%), RF: 66.3% (63.2–69.5%), as well as when clinical parameters were employed: PLS-DA: 74.1% (69.1–79.0%), RF: 65.6% (62.6–68.6%).

## Discussion

MM is a heterogeneous plasma cell dyscrasia, accounting for 1.8% of all cancer cases. Nearly all MM patients secrete monoclonal immunoglobulin, which is produced by malignant plasma cells [30, 31]. Over the last two decades, the median overall survival of MM patients has improved substantially [32, 33] due to advanced treatment options. Despite these advances, MM remains a hard-to-treat disease; most patients eventually relapse [5, 34, 35]. MM relapse is defined as the recurrence of the disease following prior treatment [11]. Due to MM heterogeneity, patients relapse from several months to years [12, 36]. Predicting the exact timing of relapse remains challenging, yet a growing body of research suggests that miRNAs have prognostic value, potentially aiding in relapse prediction [12, 34, 36].

Within the scope of this study, patients were classified into three groups (A, B, and C) based on their time to relapse. The first objective of this study was to identify differentially expressed miRNAs in PB serum among the three MM patient groups at the time of diagnosis using small RNA seq. Initial analysis of sequencing data compared group A vs. group B and group B vs. group C, revealing no statistical differences. An analysis comparing the most distant groups, A and C, revealed significant miRNA expression differences. In this comparison, 24 miRNAs were identified as differentially expressed with an adjusted *P* < 0.1; 2 miRNAs (miR-16-2-3p and miR-598-3p) showed an adjusted *P* < 0.05.

Three miRNAs (miR-16-2-3p, miR-598-3p, and miR-92b-3p), exhibited increased expression in group A compared to group C, making these two groups ideal candidates for further validation. These three miRNAs were selected for validation, confirming significant expression differences between groups A and C only for miR-598-3p (*P =* 0.0145).

The gene for miR-598-3p is located at 8p23.1. MiR-598-3p regulates gene expression associated with cellular communication, metabolic processes, and nervous system development [37]. Dysregulated expression of this miRNA is implicated in several diseases, including cancers; miR-598-3p is involved in tumor cell proliferation, apoptosis, and invasion [38]. This miRNA is overexpressed in colorectal cancer but downregulated in breast cancer or acute T lymphoblastic leukemia (T-ALL) [37, 39–41]. In T-ALL, miR-598-3p was shown to target the *DEPTOR* gene (encoding the mTOR-interacting protein with the DEP domain). Previous experiments highlighted the diverse functions of DEPTOR in cancer development. In T-ALL patients, DEPTOR promotes proliferation [41, 42]. In MM, DEPTOR was also differentially expressed; MM patients with low *DEPTOR* expression had a significantly shorter progression-free survival [43]. These studies suggest that the elevated expression of miR-598-3p, observed in group A in this study, reduces DEPTOR protein levels, potentially leading to an earlier relapse in MM patients.

The miR-15b/16 − 2 cluster is located on chromosome 3, specifically within the *SMC4* gene, which encodes structural maintenance of chromosomes 4 protein, regulating chromosome stability, assembly, and segregation through a complex with SMC2 [44]. The role of the miR-15b/16 − 2 cluster on chromosome 3 is not as well understood as the miR-15a/16 − 1 cluster, which is well-characterized in MM. Several studies suggest that the miR-15b/16 − 2 cluster plays a key role in cellular proliferation and apoptosis [44, 45]. In osteoporosis studies, miR-16-2-3p was found to target and regulate *WNT5A* expression. Protein Wnt-5a is part of the Wnt pathway, essential for bone formation and osteoblast differentiation. Studies demonstrated that increased miR-16-2-3p levels block the Wnt pathway through Wnt-5a, resulting in inhibited osteoblast differentiation, which has also been highlighted in MM studies [46–48]. Several studies have investigated this miRNA in MM patients. A study by Robak et al. on circulating serum miRNAs in MM found elevated miR-16-2-3p expression in bortezomib-resistant patients compared to bortezomib-sensitive patients [49]. Another MM study associated high miR-16-2-3p expression with poor overall survival [50]. These studies indicate that excessive miR-16-2-3p expression in MM correlates with disease aggressiveness, aligning with our findings, as miR-16-2-3p expression was elevated in group A. Dysregulation of this miRNA may be predictive for MM relapse.

MiR-92b-3p was investigated in different solid tumors and has shown context-dependent roles. It acts as a tumor-suppressive miRNA in pancreatic cancer but has an oncogenic role in colorectal, renal, breast, and prostate cancer. Its increased expression promotes cell proliferation, migration, invasion, and resistance through target gene suppression [51–56]. Increased miR-92b-3p expression predicted poor prognosis in prostate cancer patients, who exhibited shorter overall survival [56]. Breast cancer patients with high miR-92b-3p expression also showed significantly shorter relapse-free survival and overall survival [51, 54]. Since miR-92b-3p showed increased levels in group A in this study, it appears to be an oncogenic miRNA in MM and could be suitable for predicting disease relapse. Further research is needed to understand its specific role in MM.

ML algorithms have shown immense potential in enhancing the diagnosis and prognosis in MM [28, 29, 57–60]. In this study, ML models, such as RF and PLS-DA, effectively differentiated patients based on miRNA profiles, demonstrating their capability to uncover subtle patterns within complex datasets. By leveraging these algorithms, we achieved higher accuracy in distinguishing between groups A and BC. This underscores the ability of ML to augment traditional bioinformatics tools by incorporating high-dimensional data, improving predictive modeling.

The next objective of this study was to compare the mass spectra of two MM patient groups with different relapse intervals using MALDI-TOF MS; it has been used to detect peptides and proteins in PB serum in various cancers [61–63]. Several studies were conducted in MM, where MS identified potential diagnostic and classification markers [28, 29, 64–66]. For instance, our previous study showed that healthy donors were differentiated from MM patients using MALDI-TOF MS combined with artificial neural networks [24].

In this study, the OPLS-DA method assessed the data discriminatory potential, demonstrating partial separation between the groups. Four ML algorithms, namely PLS-DA, k-NN, RF, and ANN, were applied to the mass spectral data to enhance classification performance. Upon integration of clinical parameters into the dataset, significant improvements were observed in both clustering and classification accuracy. Based on the results of our study, which point to differences in the mass spectra of patients with varying relapse times in MM, comparing mass spectra and clinical parameters using MALDI-TOF MS could potentially be suitable for predicting disease relapse.

Compared to standard diagnostic tools like monoclonal protein quantification, BM biopsy or imaging, our approach uses serum-based biomarkers, enabling more frequent and minimally invasive monitoring. MALDI-TOF MS offers rapid and scalable molecular profiling, while miRNA analysis provides high specificity for early relapse prediction. Together, these methods could complement current diagnostics, addressing limitations in sensitivity and invasiveness. Analysis of circulating molecular species has become increasingly important in recent years. Our study demonstrates that while small RNA seq achieves the highest accuracy in distinguishing between groups of MM patients with different relapse timing, its routine clinical application is hindered by higher time requirements, operational costs, and technical challenges compared to MALDI-TOF MS analysis. Integrating MALDI-TOF MS analysis with clinical data emerges as a more feasible and efficient approach for relapse prediction, balancing accuracy with usability and cost-effectiveness. Nonetheless, the sample sizes for miRNA profiling and MALDI-TOF MS analyses were limited, necessitating caution in interpreting the results. Larger patient cohorts are critical for validating the identified biomarkers and ensuring their generalizability.

## Conclusions

This study investigates biomarkers for predicting early relapse in MM by analyzing circulating miRNAs and small molecular species in peripheral blood serum. Small RNA seq and subsequent RT-qPCR validation identified miR-598-3p as a specific biomarker with elevated expression in early relapse patients with high specificity. While small RNA sequencing offers high specificity in distinguishing MM relapse groups, its clinical adoption is limited due to high costs, technical demands, and processing time. MALDI-TOF MS, being fast and cost-effective, serves as a viable alternative for routine use. Integrating clinical parameters with MALDI-TOF MS spectra provides a scalable approach for rapid detection, with partial least squares discriminant analysis (PLS-DA) achieving an accuracy of 71.4%. These findings highlight the potential of liquid biopsies for early relapse prediction, with multidimensional datasets further enhancing their utility and possibly enabling timely intervention. However, the relatively small sample size used for both miRNA and MALDI-TOF MS analyses highlights the need for further investigation on larger patient cohorts to validate these findings.

## Materials and methods

### Patients’ characteristics

This study included 74 MM patients diagnosed at the University Hospital Brno, Czech Republic, between 2006 and 2021. The patients were categorized into three groups (A, B, C) based on the time of relapse after diagnosis: group A (relapse within 6 months), group B (relapse between 12 and 30 months), and group C (relapse after more than 5 years). MM patients were classified into three groups based on clinically observed relapse patterns. Group A (relapse within 6 months) reflects aggressive disease. Group C (relapse after more than 5 years) represents indolent MM with slower progression, while Group B (relapse between 12 and 30 months) encompasses an intermediate phenotype. These thresholds allowed the investigation of distinct biological mechanisms underlying relapse timing. MM samples analyzed in this study were collected at MM diagnosis. All MM patients included in this study provided informed consent, and the study was approved by the hospital’s Ethics Committee in compliance with the Declaration of Helsinki. Clinical data were obtained from the Registry of Monoclonal Gammopathies (RMG) and are summarized in Supplementary Table 1, Additional file 1. Patients received therapies per standard guidelines, with bortezomib-based regimens being predominant. Detailed therapy specifications are included in Supplementary Table 2, Additional file 1.

### Sample preparation

PB and BM samples were collected from MM patients at diagnosis. PB serum was collected as described previously [67, 68]. CD138 + PCs were isolated from BM as described previously [16].

### RNA isolation from peripheral blood serum

After thawing, PB serum samples were prepared as described before; total RNA enriched for small RNAs, including miRNAs, was isolated from 200 µl of each PB serum sample [67, 68].

### Small RNA sequencing

Small RNA seq was performed on 8 samples from each patient group (A, B, and C) to profile miRNA expression. QIAseq miRNA Library Kit (Qiagen, Germany) was used to prepare cDNA libraries, library concentration was then assessed using Qubit dsDNA HS Assay (Thermo Fisher Scientific, USA) followed by electrophoresis measurement of their size range with High Sensitivity D1000 ScreenTape Assay for Agilent 2200 TapeStation (both Agilent Technologies, USA). Equimolar amounts of cDNA libraries were pooled at a final concentration of 4 nmol·l^− 1^. Sequencing was performed using the NextSeq 500 Reagent kit v2 (Illumina, USA).

### RT-qPCR validation

To validate small RNA seq results, 7 MM samples from group A and 13 MM samples from group C were analyzed by RT-qPCR. Reverse transcription was performed with the TaqMan Advanced miRNA cDNA Synthesis Kit (Applied Biosystems, USA) using 2 µL of RNA according to the manufacturer’s instructions. RT-qPCR was performed using TaqMan Advanced miRNA Assays (Applied Biosystems, USA) as previously described [16]. The miRNA assay IDs are listed in Supplementary Table 3, Additional file 1.

### Bioinformatics and statistical analyses

The miRge3 sequencing analysis pipeline, which supports Unique Molecular Identifiers (UMI), was applied for data processing [69]. MirBase database was used for miRNA read alignment. Count-based miRNA expression data were further analyzed by R/Bioconductor libraries [70]. Only miRNAs with at least 1 read per million in at least 7 samples were analyzed. The read counts were pre-normalized by adding normalization factors within edgeR library [71, 72] and further between-sample normalized by the voom function in LIMMA library [73]. After the normalized expression levels were determined, the differentially expressed miRNAs among studied sample groups were screened applying linear model fitting and Bayes approach. The obtained P values were adjusted for multiple testing using the Benjamini–Hochberg method.

Acquired small RNA seq data were utilized to develop a predictive model for early relapse detection. Four supervised machine learning (ML) algorithms were employed: PLS-DA (partial least squares discriminant analysis), k-NN (k-nearest neighbors), RF (random forest), and ANN (artificial neural network). The caret R library was used to train and optimize the predictive model as described before [28, 29, 74].

To assess the relative expression of miRNAs, the threshold cycle values were obtained using QuantStudio 3 software (Thermo Fisher Scientific, USA). Relative miRNA expression was normalized to miR-191-5p, based on its stable expression in small RNA seq results and manufacturer’s recommendations. The relative expression was calculated by the 2^−ΔCT^ method. Normalized expression levels were analyzed using a nonparametric Mann-Whitney U test, with model accuracy assessed by Receiver Operating Characteristic (ROC) analysis.

Correlations between continuous variables were evaluated using Spearmans’ correlation coefficient. These analyses were conducted in GraphPad Prism 8.0.1 for Windows (GraphPad Software, Boston, Massachusetts USA, www.graphpad.com). Statistical significance was set at *p* < 0.05.

### Matrix-assisted laser desorption/ionization mass time-of-flight mass spectrometry (MALDI-TOF MS) sample preparation

A total of 29 PB serum samples were used for the MALDI-TOF MS analysis: 10 samples from group A and 19 samples from group BC (10 samples from group B and 9 samples from group C). Plasma samples were thawed on ice and centrifuged to remove cellular debris. Extraction involved two steps: first, 50 µL of ACN was mixed with 25 µL of plasma, sonicated, and centrifuged; the supernatant was discarded. Next, 50 µL of 50% ACN with 0.1% TFA was added to the precipitate, followed by sonication and centrifugation. The collected extract was mixed with the SA matrix in a 1:1 ratio (20 mg/mL dissolved in 50% ACN supplemented with 2.5% TFA). Immediately after, 2 µL of the homogenized sample were transferred to the metal target in five technical replicates as described previously [28].

### Acquisition of mass spectra

Mass spectra were recorded using MALDI-7090-TOF-TOF mass spectrometer (Shimadzu, Japan) equipped with a 2 kHz ultrafast solid-state UV laser (Nd: YAG: 355 nm). Mass spectra were recorded in the linear positive ion mode, in the mass region of 2–20 kDa; pulsed extraction was set to 7.5 kDa, frequency of the laser was 1 kHz, and the laser diameter was 100 μm. In total, 5 profiles from 1000 points were accumulated to record 1 mass spectrum.

### Processing of mass spectra

Raw mass spectra in the *mzML* format were preprocessed using R programming language (4.0.4) to detect differentially expressed species among mass spectra. *MALDIquant* library, and subsequently analysis using several R libraries enabling multivariate statistical modeling were used. The spectral preprocessing workflow followed standard procedures adopted from the *MALDIquant*: quality control, transformation, and smoothing, baseline correction, intensity calibration, spectra alignment, trimming (2–10 kDa), and peak detection [28, 75, 76]. The feature matrix was constructed only from the detected peaks presented in at least 20% of the total mass. An established matrix was employed in further multivariate statistical analysis and to develop a predictive ML model.

Results are presented using the orthogonal projections to latent structures discriminant analysis (OPLS-DA) using *ropls* library [28, 29]. The R2X and R2Y coefficients indicate the proportion of variance in the x and y variables that can be explained by the model. The Q2Y parameter provides an estimation of the predictive performance of the model through the 5-fold cross-validation (CV) [77].

Acquired MALDI-TOF MS data were utilized to develop a predictive ML model able to categorize patients into classes of early relapse (group A) and late-onset relapse (groups B and C combined). Furthermore, clinical parameters were added to the model to improve its predictive value. Four supervised ML algorithms were employed as described [28, 29].

## Electronic supplementary material

Below is the link to the electronic supplementary material.

Supplementary Material 1

## Data Availability

The datasets used and/or analyzed during the current study are available from the corresponding author on reasonable request.
