# Supplementary material for: Detection of early relapse in multiple myeloma patients
Source: Cell Div. 2025 Jan 29;20:4. doi: 10.1186/s13008-025-00143-3 (PMC11776158; doi:10.1186/s13008-025-00143-3)
Supplement: Supplementary file 1 — Supplementary Material 1 [file 13008_2025_143_MOESM1_ESM.docx]

**Supplementary table 1**: Baseline clinical characteristics of patients.

| **Parameters^1^** | | **Group A (N = 15)** | **Group B (N = 38)** | **Group C (N = 21)** |
| --- | --- | --- | --- | --- |
| Sex | Man | 7 | 20 | 10 |
|  | Woman | 8 | 18 | 10 |
|  | Not available | 0 | 0 | 1 |
| Age | Median  (min–⁠max) | 61  (47–⁠65) | 61  (40–⁠66) | 60  (44–⁠66) |
| Durie-Salmon stage | I | 1 | 2 | 4 |
|  | II | 4 | 5 | 3 |
|  | III | 10 | 31 | 13 |
|  | Not available | 0 | 0 | 1 |
| Durie-Salmon substage | A | 9 | 33 | 16 |
|  | B | 6 | 5 | 4 |
|  | Not available | 0 | 0 | 1 |
| ISS | 1 | 1 | 11 | 10 |
|  | 2 | 6 | 17 | 5 |
|  | 3 | 7 | 10 | 5 |
|  | Not available | 1 | 0 | 1 |
| M-protein type | IgG | 6 | 23 | 10 |
|  | IgA | 3 | 10 | 5 |
|  | IgM | 1 | 0 | 0 |
|  | Light chain only | 5 | 5 | 5 |
|  | Not available | 0 | 0 | 1 |
| Light chain type | Kappa | 8 | 22 | 13 |
|  | Lambda | 7 | 16 | 7 |
|  | Not available | 0 | 0 | 1 |
| Serum M-protein quantity (g·l^-1^) | Median (min–⁠max) | 10.4 (0.0–⁠56.8) | 36.6 (0.0–⁠89.8) | 23.2 (0.0–⁠58.2) |
| Plasmocyte count (%)  – bone marrow cytology | Median (min–⁠max) | 25.2 (3.6–⁠64.6) | 20.5 (0.0–⁠75.4) | 12.1 (0.0–⁠86.2) |
| Hemoglobin level (g·l^-1^) | Median (min–⁠max) | 91.0 (55.0–⁠130.0) | 112.0 (62.6–⁠151.0) | 117.5 (83.0–⁠150.0) |
| Thrombocyte count (10^9^·l^-1^) | Median (min–⁠max) | 153.0 (67.0–⁠301.0) | 229.5 (10.0–⁠485.0) | 245.0 (134.0–⁠749.0) |
| Calcium total level (mmol·l^-1^) | Median (min–⁠max) | 2.6 (2.0–⁠3.1) | 2.4 (2.0–⁠3.1) | 2.5 (2.0–⁠3.6) |
| Albumin level (g·l^-1^) | Median (min–⁠max) | 33.7 (24.4–⁠44.0) | 35.9 (20.8–⁠49.2) | 41.4 (29.1–⁠48.4) |
| Creatinine level (µmol·l^-1^) | Median (min–⁠max) | 149.0 (55.0–⁠540.0) | 86.0 (50.0–⁠726.0) | 85.8 (49.0–⁠639.0) |
| β2-microglobulin (mg·l^-1^) | Median (min–⁠max) | 5.5 (3.0–⁠27.4) | 4.0 (1.2–⁠50.0) | 3.1 (1.6–⁠15.8) |
| Lactate dehydrogenase (µkat·l^-1^) | Median (min–⁠max) | 3.5 (2.3–⁠11.3) | 3.0 (1.6–⁠8.8) | 3.4 (2.4–⁠5.8) |
| C-reactive protein (mg·l^-1^) | Median (min–⁠max) | 6,5 (1.0–⁠51.3) | 6.6 (0.0–⁠270.0) | 2.8 (0.9–⁠105.0) |

*^1^* Described using n in categorical variables and median (minimum–maximum) in continuous variables.

**Supplementary table 2:** Treatment regimens received by patients.

| **Treatment** | **Group A (N = 15)** | **Group B (N = 38)** | **Group C (N = 21)** |
| --- | --- | --- | --- |
| unknown | 0 | 0 | 1 |
| bortezomib + doxorubicin + dexamethason | 7 | 4 | 2 |
| bortezomib + cyklofosfamid + dexamethason | 4 | 19 | 7 |
| bortezomib + thalidomid + dexamethason | 2 | 12 | 1 |
| bortezomib + methylprednisolon | 1 | 0 | 0 |
| bortezomib + dexamethason | 0 | 1 | 0 |
| bortezomib + thalidomid + cyklofosfamid + dexamethason | 1 | 1 | 1 |
| bortezomib + thalidomid + doxorubicin + dexamethason | 0 | 1 | 1 |
| thalidomid + cyklofosfamid + dexamethason | 0 | 0 | 8 |

**Supplementary table 3:** TaqMan Advanced microRNA Assays used in the validation phase of the study.

| **miRNA** | **Assay ID** |
| --- | --- |
| hsa-miR-191-5p | 477952_mir |
| hsa-miR-16-2-3p | 477931_mir |
| hsa-miR-92b-3p | 477823_mir |
| hsa-miR-598-3p | 478172_mir |

**Supplementary table 4:** Correlation of miRNA quantity and clinical parameters.

| Parameter | Patient group | | miR-16-2-3p | | miR-92b-3p | | miR-598-3p | |
| --- | --- | --- | --- | --- | --- | --- | --- | --- |
|  |  |  | r | p | r | p | r | p |
| Age | Group A | | -0.162 | 0.733 | -0.306 | 0.512 | -0.174 | 0.750 |
|  | Group E | | -0.193 | 0.547 | -0.200 | 0.532 | 0.499 | 0.121 |
| Serum M-protein quantity (g·l^-1^) | Group A | | -0.393 | 0.396 | -0.607 | 0.167 | -0.543 | 0.297 |
|  | Group E | | -0.215 | 0.523 | -0.165 | 0.626 | 0.018 | 0.966 |
| Plasmocyte count (%) – bone marrow cytology | Group A | | 0.286 | 0.556 | 0.214 | 0.662 | 0.257 | 0.658 |
|  | Group E | | -0.196 | 0.543 | -0.119 | 0.716 | 0.218 | 0.521 |
| Hemoglobin level (g·l^-1^) | Group A | | 0.000 | 1.000 | 0.054 | 0.919 | 0.029 | 1.000 |
|  | Group E | | -0.021 | 0.956 | -0.042 | 0.904 | -0.245 | 0.468 |
| Thrombocyte count (10^9^·l^-1^) | Group A | | 0.500 | 0.267 | 0.286 | 0.556 | 0.371 | 0.497 |
|  | Group E | | -0.406 | 0.193 | -0.434 | 0.162 | 0.109 | 0.755 |
| Calcium total level (mmol·l^-1^) | Group A | | 0.179 | 0.713 | 0.536 | 0.236 | 0.486 | 0.356 |
|  | Group E | | -0.413 | 0.184 | -0.238 | 0.457 | 0.000 | 1.000 |
| Albumin level (g·l^-1^) | Group A | | 0.000 | 1.000 | -0.179 | 0.713 | 0.200 | 0.714 |
|  | Group E | | -0.441 | 0.154 | -0.531 | 0.079 | -0.555 | 0.082 |
| Creatinine level (µmol·l^-1^) | Group A | | 0.607 | 0.167 | 0.643 | 0.139 | 0.486 | 0.356 |
|  | Group E | | 0.273 | 0.391 | 0.552 | 0.067 | 0.345 | 0.299 |
| β_2_-microglobulin (mg·l^-1^) | Group A | | 0.543 | 0.297 | 0.371 | 0.497 | 0.371 | 0.497 |
|  | Group E | | **0.678** | **0.019** | **0.720** | **0.011** | 0.464 | 0.155 |
| Lactate dehydrogenase (µkat·l^-1^) | Group A | | -0.679 | 0.110 | -0.357 | 0.444 | -0.429 | 0.419 |
|  | Group E | | 0.566 | 0.059 | 0.545 | 0.071 | 0.209 | 0.539 |
| C-reactive protein (mg·l^-1^) | Group A | | -0.286 | 0.556 | 0.214 | 0.662 | -0.257 | 0.658 |
|  | Group E | | 0.042 | 0.904 | 0.231 | 0.471 | 0.309 | 0.356 |
